# Renal Oxygenation in the Pathophysiology of Chronic Kidney Disease

**DOI:** 10.3389/fphys.2017.00385

**Published:** 2017-06-28

**Authors:** Zhi Zhao Liu, Alexander Bullen, Ying Li, Prabhleen Singh

**Affiliations:** Division of Nephrology-Hypertension, University of California San Diego School of Medicine, VA San Diego Healthcare SystemSan Diego, CA, United States

**Keywords:** hypoxia, renal oxygen consumption, hypoxia inducible factor (HIF), AMPK, chronic kidney disease pathophysiology

## Abstract

Chronic kidney disease (CKD) is a significant health problem associated with high morbidity and mortality. Despite significant research into various pathways involved in the pathophysiology of CKD, the therapeutic options are limited in diabetes and hypertension induced CKD to blood pressure control, hyperglycemia management (in diabetic nephropathy) and reduction of proteinuria, mainly with renin-angiotensin blockade therapy. Recently, renal oxygenation in pathophysiology of CKD progression has received a lot of interest. Several advances have been made in our understanding of the determinants and regulators of renal oxygenation in normal and diseased kidneys. The goal of this review is to discuss the alterations in renal oxygenation (delivery, consumption and tissue oxygen tension) in pre-clinical and clinical studies in diabetic and hypertensive CKD along with the underlying mechanisms and potential therapeutic options.

## Introduction

Chronic kidney disease (CKD) is a worldwide public health problem. In the United States, 14% of adults suffer from CKD (Coresh et al., 2007). Besides its impact on health, CKD and end-stage renal disease (ESRD) require substantial healthcare resources (Honeycutt et al., 2013). Despite the resources committed to the treatment of CKD and improvements in the quality of dialysis therapy, CKD patients continue to experience significant mortality and morbidity and a reduced quality of life. Numerous studies have elucidated the underlying mechanisms of CKD and identified new therapeutic targets, but success in clinical translation has been limited. Among various pathways, imbalance between oxygen delivery and consumption leading to tissue hypoxia has been recognized as an important contributor to CKD development and progression (Fine et al., 1998; Norman and Fine, 2006; O'Connor, 2006; Mimura and Nangaku, 2010). The aim of this review is to discuss the role of renal oxygenation (oxygen delivery, consumption and tissue oxygen tension) in CKD progression and potential therapeutic options based on recent literature.

## Tubular transport

The major function of kidney is to remove waste products and excess fluid from the body to maintain homeostasis. The kidneys filter about 180 liters of fluid daily. Nearly 99% of the of filtered sodium is reabsorbed and this is the primary energy consuming process in the kidneys powered by the Na- and K-ATPase (sodium-potassium pump) in the basolateral membranes of tubular cells in the kidney. About 60–70% of total sodium reabsorption takes place in the proximal tubule, and another 25–30% in the thick ascending limb of the loop of Henle, and less than 10% in the distal convoluted tubule and collecting duct (Palmer and Schnermann, 2015). The sodium-potassium pump transports three sodium atoms out of the cell and two potassium atoms into the cell with the cost of hydrolyzing one ATP molecule (Mandel and Balaban, 1981). To retrieve nearly 99% of filtered Na, a continuously large amount of ATP (>1.7 × 10^24^ molecules) is required.

## Renal oxygenation

Both oxidative and glycolytic pathways produce ATP. Compared to 2 molecules of ATP production in glycolysis per molecule of glucose, oxidative phosphorylation, generating 30–36 molecules of ATP, is more efficient (Soltoff, 1986). Hence, sufficient oxygen delivery to the kidney is needed to generate the required ATP via mitochondrial oxidative phosphorylation. Renal blood flow in the kidney is inhomogeneous, with cortex being well-perfused, but only 10–15% of perfusion directed to medulla to preserve osmotic gradients and enhance urinary concentration (Chou et al., 1990). Moreover, the arterial-to-venous oxygen shunting in medulla, which results from a counter-current exchange of oxygen before arterial blood reaches the renal microcirculation, decreases oxygen availability in this region (Evans et al., 2008). Lastly, the high metabolic requirements of the thick ascending limbs also contribute to hypoxia particularly in the outer medullary region, where the transport-related oxygen demand is high, while the delivery is lower than the cortex (Aukland and Krog, 1960; Mandel and Balaban, 1981; O'Connor, 2006). These particular features of tubular transport and metabolism can aggravate outer medullary hypoxia.

There is a close relation between GFR, renal sodium reabsorption, and oxygen consumption (Kiil et al., 1961; Blantz et al., 2007). In most organs, an increased demand of oxygen is met by an increased blood flow to increase oxygen delivery. However, an increase in blood flow to the kidney results in a simultaneously increased tubular sodium load due to increased GFR. Since sodium reabsorption is the major determinant of renal oxygen consumption (Lassen et al., 1961; Torelli et al., 1966), increased renal blood flow besides increasing oxygen supply also increases oxygen demand attributed to increased reabsorptive sodium load. It has been shown that renal oxygen extraction remains stable over a wide range of renal blood flow (Levy, 1960), indicating the increased oxygen delivery by renal blood flow is directly counteracted by increased oxygen consumption. So, maneuvers that increase GFR and thus the tubular sodium load also increase oxygen consumption and the effect on renal oxygenation is not always predictable.

## Renal oxygenation in diabetic CKD

According to the data from National Institute of Diabetes and Digestive and Kidney Diseases, as of 2014, nearly 10% of the US population has diabetes (Diabetes-NIDDK^1^). Diabetic nephropathy develops in nearly 40% of patients with diabetes and is the leading cause of CKD in patients with ESRD (Reutens, 2013). There has been significant advances in our understanding of the various pathways in the pathogenesis of diabetic nephropathy: hyperglycemia, glomerular hyperfiltration, activation of the polyol, renin-angiotensin and the protein kinase C pathways, advanced glycation end products, and genetic susceptibility (Brownlee, 2005). However, clinical translation to successful therapeutics for diabetic nephropathy has been limited. Since Fine et al. proposed kidney hypoxia as a mediator of progressive kidney disease (Fine et al., 1998), numerous experimental and clinical studies have provided evidence in support of chronic hypoxia being a common pathway for various progressive kidney diseases including diabetic nephropathy (Nangaku, 2006; Palm, 2006; Singh et al., 2008a, 2013; Mimura and Nangaku, 2010; Palm and Nordquist, 2011).

In experimental streptozotocin-induced diabetes, Ries et al. demonstrated that hypoxia was present in all compartments of the diabetic kidney, particularly in the outer medulla, using blood oxygen level-dependent (BOLD) MRI and diffusion-weighted imaging (Ries et al., 2003). Palm et al. showed a decrease in renal oxygen tension in the diabetic kidney, particular in medulla, due to the augmented oxygen consumption (Palm et al., 2003). Intrarenal microcirculation was not changed, but deoxyhemoglobin signals rose in diabetic kidney and oxygen consumption by tubular cells was significantly enhanced (Palm et al., 2003, 2004). Later the same group confirmed their findings using BOLD imaging (Edlund et al., 2009). Another study, demonstrated that hypoxic changes can be detected as early as 2 days in the diabetic kidney (dos Santos et al., 2007). Subsequently, Rosenberger et al. provided information on cellular localization of hypoxia in thick ascending limbs and a lesser extent in the collecting ducts using immunohistochemical staining with pimonidazole, a molecular hypoxia probe, and hypoxia inducible factor (HIF) (Rosenberger et al., 2008).

Recent studies have demonstrated the presence of tissue hypoxia before the presence of markers of kidney injury in diabetes. Friederich-Persson et al. reported kidney hypoxia resulting from increased mitochondrial oxygen consumption, independent of hyperglycemia and oxidative stress, was associated with tubulointerstitial damage, proteinuria and infiltration of inflammatory cells (Friederich-Persson et al., 2013). They concluded that kidney tissue hypoxia, *per se*, may be sufficient to initiate the development of nephropathy. Others have demonstrated the presence of tissue hypoxia in the kidney before the onset of albuminuria in diabetic mice (Franzén et al., 2016). Functional MRI imaging techniques have also been used to assess intrarenal oxygenation in humans, specifically in diabetic patients. Inoue et al. demonstrated parenchymal hypoxia and fibrosis in the renal cortex of CKD patients with or without diabetes using BOLD MRI and diffusion-weighted MRI, respectively (Inoue et al., 2011). Yin et al. reported reduced cortical and medullary oxygenation in patients with type 2 diabetes with nephropathy (Yin et al., 2012). Medullary hypoxia was more prominent and present at earlier stages, with cortical hypoxia becoming more apparent with worsening renal function in diabetic patients. Pruijm studied the role renin-angiotensin system (RAS) in renal oxygenation in early stage type 2 diabetic patients with evidence of nephropathy (albuminuria and/or hypertension) (Pruijm et al., 2013). Short-term RAS blockade with ace-inhibitor or angiotension receptor blocker did not increase renal tissue oxygenation. Although not specifically examined in diabetes, animal studies have demonstrated an increase renal oxygenation in both normal and remnant rat kidney with RAS inhibition (Norman et al., 2003; Deng et al., 2009). Short duration of RAS blockade or modest changes in oxygenation not detected by BOLD MRI may explain the discrepancy.

Other studies have examined renal oxygenation in diabetes before any evidence of nephropathy and found similar baseline renal oxygenation in cortical and medullary oxygenation in diabetes compared to controls with BOLD MRI (Epstein et al., 2002; Economides et al., 2004). However, remarkably in both studies, patients with diabetes and even those at risk of diabetes did not show any improvement in medullary oxygenation with water diuresis, which was observed in healthy controls. This suggests an early impairment of adaptive vasodilation in the renal medulla in diabetes. Conversely, in a study in patients with moderate to severe diabetic nephropathy, no differences in cortical oxygenation but higher medullary oxygenation compared to controls was observed (Wang et al., 2011). The authors speculate that hypoxia noted in other studies in early stages of diabetes may be due to high oxygen consumption to support increased tubular reabsorption during the stage of glomerular hyperfiltration. In their study, reduced GFR and the associated decrease in tubular sodium reabsorption may result in lower oxygen consumption and the apparent increase in medullary oxygenation. The other possibility may be related to the technical aspects of BOLD MRI measurements. BOLD MRI measures tissue oxygenation indirectly by the changes in the deoxyhemoglobin concentration in the capillaries supplying the tissue. In advanced nephropathy, tissue fibrosis, reduction in peritubular capillaries, and changes in tissue oxygen extraction may lead to discrepancies between capillary deoxyhemoglobin concentrations and tissue oxygenation. Technical aspects of BOLD MRI and standard protocols for these measurements have been extensively discussed in recent papers (Simon-Zoula et al., 2006; Pruijm et al., 2016; Hirakawa et al., 2017). Characteristics of the studies discussed is summarized in Table 1.

**Table 1:** Characteristics of studies in diabetes/diabetic CKD.

**Species**	**Types of diabetes**	**Duration of diabetes**	**Blood glucose (mmol/l)**	**HbA_1c_ (%)**	**GFR (ml/min)**	**eGFR (ml/min/1.73 m^2^)**	**Urinary protein (mg/g uCr)**	**References**
Rat	STZ induced diabetes	Up to 3 weeks	47.8 ± 14		N/A		N/A	Ries et al., 2003
		14 days	20.9 ± 1.0					Edlund et al., 2009
		Up to 4 weeks	24.5 ± 1.7					dos Santos et al., 2007
		4 weeks	25.5 ± 1.2		1.14 ± 0.06			Palm et al., 2003
		4 weeks	25.1 ± 0.6		1.01 ± 0.08			Palm et al., 2004
		Up to 90 days	23.0 ± 1.5		≤ 1.97 ± 0.1			Rosenberger et al., 2008
Mouse	Alloxan induced diabetes	Up to 15 days	23.8 ± 1.7		N/A		Negative	Franzén et al., 2016
Human	Diabetes^*^	N/A	N/A	7.0 ± 1.2		43.8 ± 27.7	980.0 ± 2229.1	Inoue et al., 2011
	Type 2 diabetes	5.6 ± 5.5 years	7.8–11.1	7.7 ± 1.8		N/A	30-300	Economides et al., 2004
		7 ± 4.75 years	8.4 ± 1.0	7.4 ± 0.46	133 ± 7		Negative	Epstein et al., 2002
		7.85 ± 5.31 years	N/A	N/A	≤ 88.45		≤ 9989.55	Yin et al., 2012
						±22.29	±7421.91	
		11 ± 7 years	7.2 ± 3	7.8 ± 1.0	62 ± 22		Positive	Pruijm et al., 2013
	Diabetes^*^	N/A	N/A	N/A		≤ 67	Positive	Wang et al., 2011

*CKD, chronic kidney disease; STZ, streptozotocin; HbA1c, hemoglobin A1c; GFR, glomerular filtration rate; eGFR, estimated GFR; uCr, urinary creatinine; N/A, information not available*.

* *Diabetes type not specified*.

## Renal oxygenation in hypertension and hypertensive CKD

Nearly 30% of adults in the United States hypertension according to the National Health and Nutrition Examination Survey 2011–2012 (Ostchega et al., 2008). Hypertension can affect each renal compartment: vessels, glomeruli and tubulointerstitium (Meyrier, 2015). Hypertensive nephropathy is the second only to diabetic nephropathy as the most common cause of ESRD in the US (Udani et al., 2011). Franz Volhard and Theodor Fahr first introduced the term hypertensive nephrosclerosis in 1918 (Meyrier, 2015) and several studies investigating the pathophysiology and potential treatments have been published. Over the last two decades, the role of renal oxygenation in hypertensive nephrosclerosis has been investigated.

Welch et al. were the first to show that a lower tissue oxygenation in both the cortex and medulla in angiotensin II (Ang II)-infused or spontaneously hypertensive rats (Welch et al., 2001, 2003). They further demonstrated that oxygenation was restored with the administration of superoxide dismutase mimetic, tempol, or an angiotensin receptor blocker, candesartan. In spontaneously hypertensive rats, they also observed a reduction in the ratio of tubular sodium transport by oxygen consumption (TNa/QO2). In an acute hypertension model with intravenous infusion of Ang II, reduced the oxygenation in the cortex and isolated proximal tubules and reduced TNa/QO2 was observed (Welch et al., 2005). All the effects were reversed by tempol, demonstrating the role of oxidative stress in this model. Recently, Emans et al. studied the effects of Ang II on cortical pO2 by telemetry in conscious rats (Emans et al., 2016). Using exogenous Ang II intravenously or activated RAS in transgenic Cyp1a1Ren2 rats, they found reduced renal cortical PO2 preceding the development of tissue injury. Zhu et al. showed that chronic infusion of Ang II in uninephrectomized rats increased tissue hypoxia as measured by immunostaining with pimonidazole, as well as increased urinary albumin excretion and collagen accumulation (Zhu et al., 2011). The albuminuria and collagen accumulation was attenuated by gene silencing of HIF-1α, linking upregulation of HIF-1α with fibrosis in this model. In another model of hypertension, renal neurogenic hypertension, induced by intrarenal injection of phenol, was shown to renal increase HIF-1α expression (Koeners et al., 2014). Interestingly, pretreatment with cobalt chloride, known HIF-1α activator, attenuated the development of hypertension and renal vasoconstriction.

Subtotal nephrectomy (STN), by uninephrectomy and partial renal infarction in the contralateral kidney, is a well-established, representative model of hypertensive CKD. Increased oxygen consumption factored for sodium reabsorption or nephron number has been observed in this model. Harris et al. observed a threefold increase in oxygen consumption per nephron in STN kidney at 4 weeks (Harris et al., 1988). Similar results were observed by Nath et al. at 3 weeks in the STN kidney (Nath et al., 1990). We have extensively studied the early hemodynamic, transport and metabolic adaptations in the 1-week STN kidney (Deng et al., 2009, 2010; Singh et al., 2009, 2012; Singh and Thomson, 2014). A major adaptation to nephron loss in early STN is hyperfiltration in the remaining nephrons (Singh et al., 2009). Hyperfiltration in the remaining nephrons results in hyperreabsorption, and this is associated with increased oxygen consumption. Indeed, we have previously reported the high oxygen consumption factored for sodium reabsorption at 1 week after STN (Deng et al., 2009, 2010). We have also demonstrated improved renal oxygenation with RAS blockade and HIF-1α activation (Deng et al., 2009, 2010). Manotham et al. showed evidence of tissue hypoxia in the STN kidney as early as 4 days and more prominently at 7 days by using pimonidazole immunostaining (Manotham et al., 2004). Tissue hypoxia persisted until interstitial damage developed. Treatment with Ang II blocker, olmesartan, prevented vascular changes and ameliorated tubular hypoxia after nephron loss.

In terms of clinical evidence, Textor et al. showed that African-American, hypertensive patients had elevated renal medullary volumes and blood flow, and lower medullary oxygenation compared to white hypertensive patients (Textor et al., 2012). Pruijm et al. investigated the effect of sodium intake on renal oxygenation in normotensive and hypertensive subjects after 1 week of high sodium and 1 week of low-sodium diet using BOLD MRI (Pruijm et al., 2010). They found that low sodium intake was associated with an increased renal medullary oxygenation but no changes in renal cortical oxygenation in both normo- and hypertensive individuals. In hypertensive patients, medullary oxygenation correlated with 24-h sodium excretion but no correlation with segmental renal sodium reabsorption was seen as observed in normotensive group. They speculated that this may be due to enhanced proximal sodium reabsorption and salt sensitivity observed in hypertensive patients (Burnier et al., 2006).

Schachiger et al. studied the role of RAS activation in renal oxygenation in humans and showed an acute decrease in cortical oxygenation by BOLD MRI after Ang II bolus injection (Schachinger et al., 2006). Subsequently, another study demonstrated a dose-dependent decrease of renal blood flow accompanied by a minor decrease of oxygenation in the cortex, not in the medulla with continuous Ang II infusion in healthy humans (Bel et al., 2016). As discussed above, parenchymal hypoxia and fibrosis in the renal cortex of CKD patients using BOLD MRI and diffusion-weighted MRI has been shown (Inoue et al., 2011). Other recent studies have demonstrated increased renal oxygenation, particularly in the renal medulla, with RAS blockade in healthy subjects and in CKD patients (Stein et al., 2012; Siddiqi et al., 2014; Vink et al., 2015). Characteristics of the studies discussed is summarized in Table 2.

**Table 2:** Characteristics of studies in hypertension/hypertensive CKD.

**Species**	**Model/type of hypertension**	**Duration of hypertension**	**GFR (ml/min)**	**eGFR (ml/min/1.73 m^2^)**	**Urinary protein (mg/d)**	**References**
Rat	STN	Up to 1 week	N/A		≤ 51.5 ± 8.6	Manotham et al., 2004
		1 week	0.53 ± 0.04		N/A	Deng et al., 2010
		8 days	0.85 ± 0.23			Singh et al., 2009
		8 days	1.05 ± 0.20 (low salt) 0.95 ± 0.19 (high salt)			Singh and Thomson, 2014
		3 weeks	0.66 ± 0.07			Nath et al., 1990
	STN, unilateral nephrectomy	1 week	N/A			Singh et al., 2012
	Drug-induced	19 days	No change compared to control			Emans et al., 2016
Human	Essential	N/A		95 ± 24	120 ± 293	Textor et al., 2012
	Essential	N/A		106.9 ± 15.6	Negative	Pruijm et al., 2010
	Drug-induced	1 day		N/A	Negative	Schachinger et al., 2006
	Drug-induced	1 day		110 ± 18	N/A	Bel et al., 2016
	Essential and secondary	N/A		30 ± 11	N/A	Siddiqi et al., 2014
	Essential	N/A		75	2.2 mg/mmol, albumin/creatinine ratio	Vink et al., 2015

*CKD, chronic kidney disease; STN, subtotal nephrectomy; GFR, glomerular filtration rate; eGFR, estimated GFR; N/A, information not available*.

## Pathophysiology of hypoxia in CKD

Tubulointerstitial injury, which encompasses tubular atrophy and interstitial fibrosis, is a hallmark of progressive CKD regardless of its cause. Several studies have validated the role of tissue hypoxia in the progression of kidney disease (Kang et al., 2002; Nangaku, 2006; Heyman et al., 2008; Singh et al., 2013). Compromised oxygen delivery due to structural and functional changes impairing blood flow has been demonstrated. Tubulointerstitial hypoxia in advanced CKD can result from glomerular injury and interstitial fibrosis (Heyman et al., 2008). Glomerulosclerosis decreases the blood flow to downstream peritubular capillaries leading to insufficient oxygen delivery. Interstitial fibrosis further impairs oxygen delivery from the peritubular capillaries to the tubules. Recent studies have demonstrated peritubular capillary dysfunction as an important factor in the initiation of renal hypoxia (Ohashi et al., 2000; Kang et al., 2002; Matsumoto et al., 2004; Kawakami et al., 2014). Animal and human biopsy specimens in CKD have demonstrated that tubulointerstitial injury is preceded by rarefaction of the peritubular capillaries (Bohle et al., 1996; Choi et al., 2000; Babickova et al., 2017).

The significance of these structural changes aside, early hypoxia demonstrated in animal and human studies are likely due to altered hemodynamics and metabolism. As discussed above, alteration in renal oxygen consumption and tissue hypoxia has been demonstrated in both diabetic and non-diabetic CKD models prior to the development of structural changes (Ries et al., 2003; Manotham et al., 2004; dos Santos et al., 2007; Deng et al., 2009, 2010; Franzén et al., 2016). Moreover, kidney hypoxia resulting from increased mitochondrial oxygen consumption alone has been shown to induce kidney injury (Friederich-Persson et al., 2013). Hence, understanding of the determinants and regulation of renal oxygenation in early stages is valuable in preventing subsequent tissue injury.

Chronic hypoxia causes tissue damage via various cytokine and cell-signaling pathways including RAS, endothelin, plasmin activator inhibitor-1, adhesion molecules and growth factors (Heyman et al., 2008; Haase, 2013). Impaired nitric oxide availability has also been implicated in the pathophysiology of diabetic nephropathy (Palm et al., 2003; Prabhakar et al., 2007) and hypertensive nephropathy (Welch et al., 2003; Singh et al., 2008b; Deng et al., 2009). Nitric oxide can increase renal oxygenation by improving oxygen delivery via vasodilation and directly lowering oxygen consumption (Evans and Fitzgerald, 2005; Singh et al., 2008b). HIF, a key transcriptional regulator of cellular adaption to hypoxia, has also received significant attention. It induces various target genes that impact oxygen delivery via vasomotor regulation and cellular oxygen consumption via several pathways (Semenza, 2009). Downstream HIF target proteins include inducible NOS, heme oxygenase-1, vascular endothelial growth factor, glucose transproters, glycolytic enzymes as well as proteins involved in cell proliferation and survival (Nangaku and Eckardt, 2007; Gunaratnam and Bonventre, 2009). HIF activation induces a glycolytic phenotype by upregulating GLUT transporters to facilitate glucose entry and inducing glycolytic enzymes (Semenza, 2009). HIF also has significant effects on mitochondrial metabolism and improves efficiency of respiratory chain proteins in hypoxia (Wheaton and Chandel, 2011). It represses mitochondrial biogenesis and respiration (Papandreou et al., 2006). It also induces mitochondrial autophagy as an adaptive metabolic response in hypoxia (Zhang et al., 2008).

While HIF activation has been shown to be renoprotective role in acute kidney injury, its effect in CKD are conflicting (Nangaku and Eckardt, 2007; Haase, 2009). Pharmacological HIF activation improved renal oxygenation, function and morphology in streptozotocin-induced diabetic and in STN rats (Deng et al., 2010; Li et al., 2015; Nordquist et al., 2015). Other studies have also shown beneficial effects of systemic HIF activation with different pharmacological agents in experimental models characterized by hypoxia (Manotham et al., 2004; Tanaka et al., 2005; Song et al., 2010; Yu et al., 2012a). However, studies using genetic approaches have yielded conflicting results. HIF-1α gene silencing or HIF-1α knockout has been shown to attenuate albuminuria, collagen accumulation and the development of tubulointerstitial fibrosis (Welch et al., 2005; Wang et al., 2014). Proximal tubule specific overexpression of HIF-1α under normoxic conditions increased fibrosis while ablation ameliorated it (Higgins et al., 2007, 2008). Interestingly, the same group has subsequently shown that global activation of HIF-1α in all tubules ameliorated inflammation and fibrosis (Kobayashi et al., 2012). These results suggest that the outcomes of HIF activation are cell-specific, context-specific and timing-dependent in CKD (Yu et al., 2012b).

**Figure 1:**
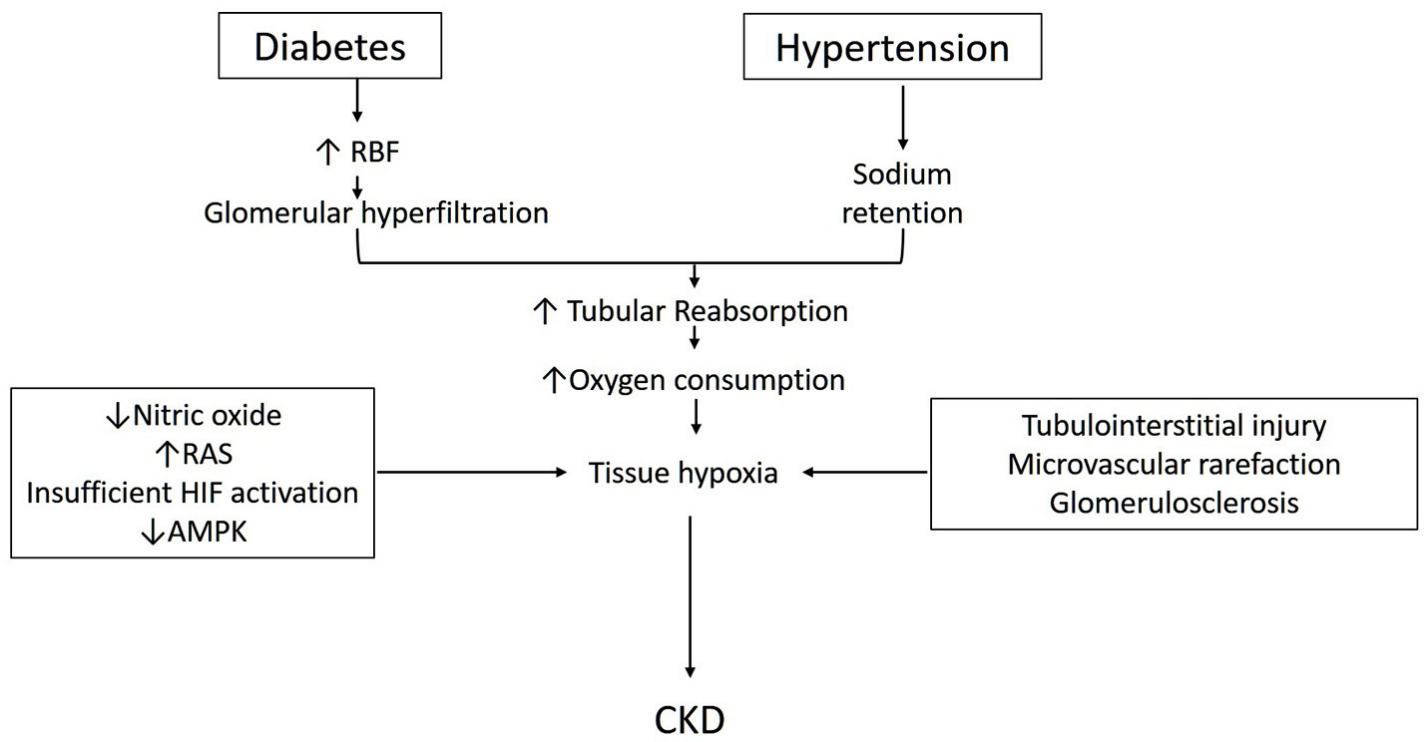
Schematic describing pathophysiology for the development of CKD in diabetes and hypertension (see text for details).

Lastly, the role AMP-activated kinase in the metabolism of diabetic and non-diabetic CKD is also receiving attention. AMPK is a ubiquitously expressed, highly conserved, key energy sensor and regulator of cellular metabolic activity (Hardie and Ashford, 2014). It is activated by cellular stressor such as nutrient deprivation, hypoxia or ischemia. AMPK facilitates metabolic adaptation by triggering ATP producing pathways, while inhibiting ATP consuming pathways (Gwinn et al., 2008). It stimulates mitochondrial biogenesis and cellular autophagy as survival mechanisms in low energy states (Steinberg and Kemp, 2009; Weinberg, 2011). Beneficial effects of AMPK activation in diabetes and high-fat diet induced kidney disease has been described (Lee et al., 2007; Hallows et al., 2010; Decleves et al., 2011). AMPK activation has also been shown to improve oxygen delivery and lower oxygen consumption and improve renal function and histology in STN (Satriano et al., 2013). We have also recently described the interactions between HIF and AMPK pathways in the regulation of cellular hypoxia adaptation in STN (Li et al., 2015). A schematic diagram incorporating some of the above discussed aspects in the pathophysiology of development of CKD is shown in Figure 1.

## Conclusion

CKD is a major healthcare burden, associated with significant comorbidities and progression to ESRD. Available therapies for diabetic and non-diabetic CKD are inadequate in halting or slowing the progression of disease. An increasing number of experimental and clinical studies indicate that renal tissue hypoxia, resulting from mismatch between oxygen delivery and consumption is a major contributor to CKD progression. Hence, it is imperative to understand the regulation of kidney oxygenation in CKD to identify novel therapeutic targets. Interventions to restore kidney tissue oxygenation has been shown to be beneficial in animal models of CKD. Clinical studies are limited and additional investigations into therapeutic targets to improve renal oxygenation in different forms of CKD are needed to improve clinical outcomes.

## Author contributions

ZZL: drafted, prepared, and edited the manuscript. AB and YL: reviewed and edited the manuscript. PS: reviewed, revised and edited the manuscript.

### Conflict of interest statement

The authors declare that the research was conducted in the absence of any commercial or financial relationships that could be construed as a potential conflict of interest.

## Footnotes

^1^Diabetes-NIDDK. National Institute of Diabetes and Digestive and Kidney Diseases. https://wwwniddknihgov/health-information/diabetes (accessed 2017).
